# Spatial Contributions to Nuclear Magnetic Shieldings

**DOI:** 10.1021/acs.jpca.0c10884

**Published:** 2021-02-19

**Authors:** Rahul Kumar Jinger, Heike Fliegl, Radovan Bast, Maria Dimitrova, Susi Lehtola, Dage Sundholm

**Affiliations:** † Indian Institute of Science Education and Research, Dr. Homi Bhabha Road, Pashan, Pune 411008, India; ‡ Karlsruhe Institute of Technology, Institute of Nanotechnology, Hermann-von-Helmholtz Platz 1, D-76344 Eggenstein-Leopoldshafen, Germany; § Department Information Technology, UiT Arctic University Norway, Tromsø, Norway; ∥ Department of Chemistry, Faculty of Science, University of Helsinki, P.O. Box 55, FI-00014 Helsinki, Finland; ⊥ Molecular Sciences Software Institute, Blacksburg, Virginia 24061, United States

## Abstract

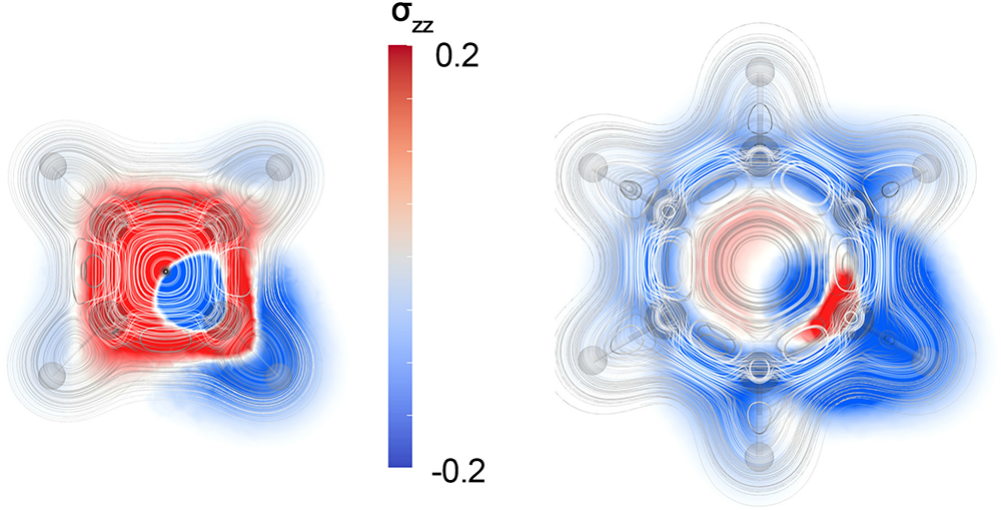

We develop a methodology for calculating, analyzing, and visualizing nuclear magnetic shielding densities which are calculated from the current density via the Biot–Savart relation. Atomic contributions to nuclear magnetic shielding constants can be estimated within our framework with a Becke partitioning scheme. The new features have been implemented in the GIMIC program and are applied in this work to the study of the ^1^H and ^13^C nuclear magnetic shieldings in benzene (C_6_H_6_) and cyclobutadiene (C_4_H_4_). The new methodology allows a visual inspection of the spatial origins of the positive (shielding) and negative (deshielding) contributions to the nuclear magnetic shielding constant of a single nucleus, something which has not been hitherto easily accomplished. Analysis of the shielding densities shows that diatropic and paratropic current-density fluxes yield both shielding and deshielding contributions, as the shielding or deshielding is determined by the direction of the current-density flux with respect to the studied nucleus instead of the tropicity. Becke partitioning of the magnetic shieldings shows that the magnetic shielding contributions mainly originate from the studied atom and its nearest neighbors, confirming the localized character of nuclear magnetic shieldings.

## 1 Introduction

Second-order magnetic properties such as nuclear magnetic shieldings, indirect spin–spin coupling constants, and magnetizabilities are usually calculated by using the gradient theory of electronic structure calculations as the second derivative of the electronic energy with respect to the external magnetic perturbation(s) in the limit of vanishing perturbation(s).^1−4^ However, the elements of the nuclear magnetic shielding and magnetizability tensors can also be obtained as second derivatives of the magnetic interaction energy, which can be written as an integral over the scalar product of a current density caused by a magnetic perturbation and the vector potential of the second magnetic perturbation.^5−7^ The current density **J**^**B**^(**r**) induced by an external magnetic field **B**—or the current density  induced by the nuclear magnetic moment **m**_*I*_ of nucleus *I*—is formally defined as the real part  of the mechanical momentum density
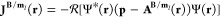
1where **p** = −i∇ is the momentum operator and Ψ(**r**) is a complex wave function because of the vector potential of the magnetic perturbation **A**^**B**^(**r**) or . As will be discussed in later in this work, the magnetic properties evaluated within this scheme have no reference to the magnetic gauge origin if the current density is gauge origin independent, as is the case in our GIMIC approach^8−11^ as well as in the ipsocentric approach.^12−16^

While the end results of the gradient-theory and the integration approaches are the same, the method based on integration can be used for providing additional information about orbital and spatial contributions to a given magnetic property. For instance, magnetizabilities, which are usually calculated using gradient theory as the second derivative of the electronic energy with respect to the external magnetic field, can also be obtained as the second derivative of the magnetic interaction energy expressed in terms of the current density induced by the magnetic field^17−19^
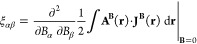
2As we have recently discussed in ref (19), eq 2 can be used to extract information about the spatial contributions to components of the magnetizability tensor.

The nuclear magnetic shielding tensor for nucleus *I*, in turn, is determined by the second derivative of the magnetic interaction energy with respect to the external magnetic field **B** and the nuclear dipole moment **m**_*I*_. The shielding tensor can then be calculated from the current density induced by the external magnetic field **J**^**B**^(**r**) and the vector potential of the nuclear magnetic moment 
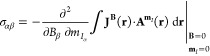
3Alternatively, the shielding tensor can be calculated from the current density induced by the nuclear magnetic moment and the vector potential of the external magnetic field **A**^**B**^(**r**) and the current density induced by the nuclear magnetic moment  of nucleus *I*(5,17,18,20)
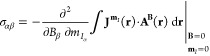
4Equation 3 is typically used in computations since picking the expression with **J**^**B**^(**r**) means that the current density has to be computed only for the three components of the external magnetic field instead of the 3*N* components of the magnetic dipole moments of *N* nuclei. However, efficient algorithms have also been developed by using eq 4, as the localized nature of the current densities induced by nuclear magnetic moments allows for powerful use of screening and parallelization.^21,22^

Because the current density **J**^**B**^(**r**) induced by an external magnetic field is a function of the strength of the external magnetic field, differentiation of the magnetic interaction energy yields the first derivative of the current density with respect to the external magnetic field (∂**J**^**B**^(**r**)/∂**B**), which is the current-density susceptibility tensor (CDT) induced by the external magnetic field.^18,23^ Analogously, the differentiation with respect to the nuclear magnetic moment acts only on the vector potential of the nuclear magnetic moment, yielding , since only that term in eq 3 depends on the nuclear magnetic moment. The dot product of these two quantities, ∂**J**^**B**^(**r**)/∂**B** and , is a scalar function known as the nuclear magnetic shielding density.^5−7^ The spatial distribution of the shielding density provides detailed information about the origin of the individual elements of the nuclear magnetic shielding tensor as well as the shielding constants.^15,24−28^

Further information about the magnetic shielding density can be obtained from the individual orbital contributions to the magnetic shieldings^15^ and shielding functions. Dividing the magnetic shielding density into positive and negative parts as well as into orbital contributions shows the spatial origins of the shielding and deshielding contributions to the shielding tensor and the isotropic shielding constants.^27,29^ Thus, calculations of magnetic shielding densities provide a rigorous physical basis for interpreting nuclear magnetic resonance (NMR) chemical shifts.

In this work, we develop a methodology for analyzing spatial contributions to nuclear magnetic shielding constants. We apply the methods to the hydrogen and carbon nuclei in benzene (C_6_H_6_) and cyclobutadiene (C_4_H_4_), which are test cases representing aromatic and antiaromatic hydrocarbons, respectively. Next, we present the underlying theory in section 2.1 and continue in section 2.2 with the employed numerical methods. Then, in section 2.3, we describe the computational methods. We discuss the magnetic shielding densities of the studied molecules in section 3 and summarize our study and form our main conclusions in section 4.

## 2 Methods

### 2.1 Theory

The vector potential  in international standard (SI) units arising from the nuclear magnetic dipole moment **m**_*I*_ of nucleus *I* can be chosen as
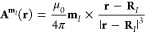
5where **R**_*I*_ is the position of the *I*th nucleus and μ_0_ is the vacuum permeability.^30^ Similarly, the vector potential **A**^**B**^(**r**) of an external static magnetic field is
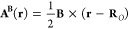
6where **R**_*O*_ is the chosen magnetic gauge origin. The magnetic flux density **B** and the magnetic dipole moment **m**_*I*_ are uniquely defined by the vector potentials **A**^**B**^(**r**) and , whereas the reverse does not hold since all the vector potentials of the form **A**′ = **A** + ∇*f*(**r**) generate the same magnetic field **B**, as ∇ × ∇*f*(**r**) = **0** for any smooth function *f*(**r**).

Even though exact solutions of the Schrödinger equation are gauge invariant, the use of finite one-particle basis sets introduces a gauge dependence in quantum chemical calculations of magnetic properties. The CDT can be made gauge origin independent by using gauge-including atomic orbitals (GIAOs), also called London atomic orbitals (LAOs). The GIAOs are defined as^8,31,32^
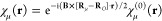
7where i is the imaginary unit and χ_μ_^(0)^(**r**) is a standard Gaussian-type basis function centered at **R**_μ_. The use of GIAOs eliminates the gauge origin from the expression we use for calculating the CDT (∂*J*_α_^**B**^(**r**)/∂*B*_β_):^8,10,11^
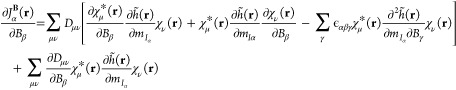
8In eq 8, **D** is the density matrix in the atomic orbital basis, ∂**D**/∂**B** are the magnetically perturbed density matrices, ϵ_αβγ_ is the Levi–Civita symbol, *h̃*(**r**) denotes the magnetic interaction operator without the |**r** – **R**_*I*_|^–3^ denominator with
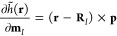
9and
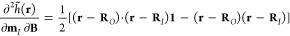
10and **R**_*I*_ is the position of nucleus *I*. Finally, the nuclear magnetic shielding tensor of nucleus *I*, σ_αβ_^*I*^, can be calculated from eqs 3 and 5 as
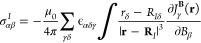
11It is important to note that all terms that contain the gauge origin **R**_*O*_ cancel in eq 8, making the CDT calculation as well as eq 11 independent of the gauge origin. Analogously, all terms in eq 8 containing the nuclear position **R**_*I*_ also cancel, eliminating explicit references to the coordinates of the nucleus *I* from the current density (the physical implicit dependence still remains). As a result, the integrated second-order magnetic properties have no reference to the gauge origin or the nuclear coordinates.

The Biot–Savart expression in eq 11 has advantages over the corresponding second-derivative expression. Contributions to the tensor elements can be visually interpreted by plotting the positive and negative parts of the integrand separately, yielding information about shielding and deshielding contributions to the elements of the magnetic shielding tensor. For example, in a system with a ring current, the σ_*zz*_^*I*^ contribution given by
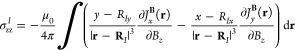
12will consist of both positive and negative shielding contributions due to the relative direction of the current density with respect to the investigated atom *I*.^11,33^

The Biot–Savart expression in eq 11 can be calculated by quadrature when the CDT is known. Because established gradient-theory implementations of NMR shielding constants are typically used to compute the CDT, the shielding constants from eq 11 do not provide any new physical information; however, the numerically evaluated shielding constants can be compared to the analytically evaluated values to assess the accuracy of the numerical integration of the Biot–Savart expressions, which is useful for applications to other second-order magnetic properties. For instance, a similar approach has recently been used to calculate and assess the accuracy of magnetizabilities from new density functional approximations, even though analytical methods to calculate the magnetizability tensor were not available in the used program.^19^

### 2.2 Implementation

A numerical integration scheme for calculating spatial contributions to nuclear magnetic shieldings has been implemented into the freely available GIMIC program.^34^ The atomic contributions to the magnetic shieldings are obtained by quadrature over atomic domains generated by the NUMGRID library,^35^ which is based on the use of Becke’s multicenter scheme.^36^ The atomic domains were determined with the Becke partitioning scheme,^36^ employing the iteration order *k* = 3 in the construction of the cutoff function as suggested by Becke. The radial integration points of the atom-centered grids are generated as suggested by Lindh et al.,^37^ and Lebedev’s angular grids are used.^38^ The CDT is constructed in GIMIC with eq 8 from the density matrix, the magnetically perturbed density matrices, and basis set information obtained from Turbomole^39^ calculations of NMR shielding constants.

### 2.3 Computational Methods

The molecular structures of C_6_H_6_, C_4_H_4_, and B_3_N_3_H_6_ were optimized with Turbomole^39^ version 7.5 employing the B3LYP density functional,^40−42^ the def2-TZVP basis set,^43^ and the m5 quadrature grid;^44,45^ the optimized molecular structures are given in the Supporting Information. Nuclear magnetic resonance (NMR) shielding constants were also calculated with Turbomole at the same level of theory by using GIAOs.^31,32,46,47^ In the NUMGRID calculations, 21042 grid points were used for each carbon and 19234 grid points for each hydrogen.

The B3LYP/def2-TZVP level of theory has been found to yield good agreement compared to second-order Møller–Plesset (MP2) theory for the ^1^H NMR magnetic shielding in tetramethylsilane (TMS, Si(CH_3_)_4_), as the ^13^C shielding in TMS reproduced by the method deviates by only 7% (roughly 12 ppm) from the one obtained at the MP2/def2-TZVP and MP2/def2-TZVPP levels of theory.^48^ Although we are aware that these results are not fully converged to the complete basis set limit, especially for the carbon shieldings,^49^ the B3LYP/def2-TZVP level of theory suffices for our present purposes of illustrating the spatial origins of magnetic shieldings: the accurate reproduction of ^13^C shieldings is known to be challenging,^50^ and the functional error is likely of the same order of magnitude as the basis set truncation error.

The methods presented in section 2.2 and their GIMIC implementation, however, can be applied in combination with any basis set or level of theory for which the density and perturbed density matrices are available. Basis set truncation errors for the def2-TZVP shieldings and their effects on the atomic contributions will be discussed in section 3.3, showing that the truncation errors in def2-TZVP only affect the contribution to the shielding of the same atom, whereas the contributions to the shieldings of the other atoms are reproduced accurately in the def2-TZVP basis set.

## 3 Results and Discussion

### 3.1 Benzene

**Figure 1:**
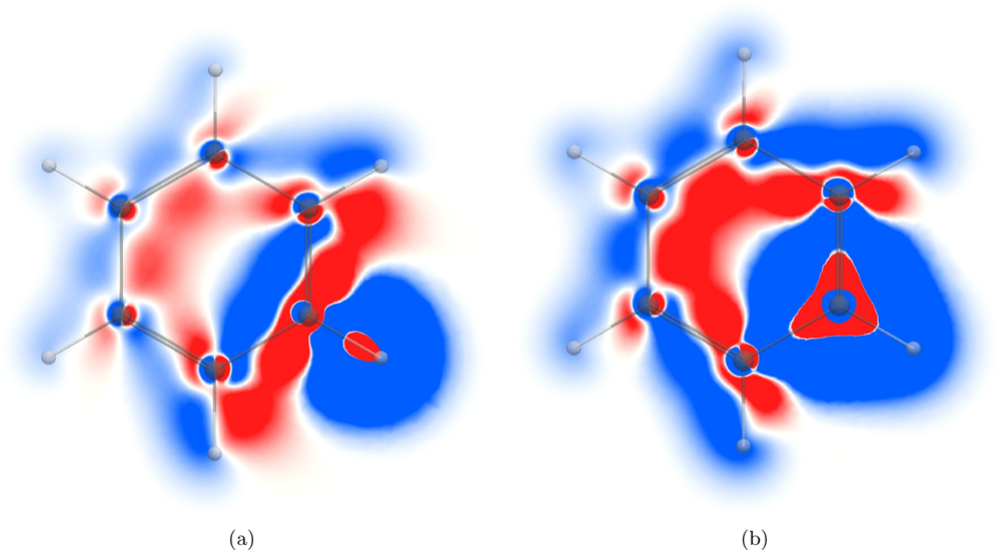
*zz* component of the magnetic shielding density of the (a) ^1^H NMR shielding and (b) ^13^C NMR shielding in the molecular plane of C_6_H_6_. The shielding contributions are shown in blue and the deshielding contributions in red in the range [−0.2; 0.2].

The magnetic shielding density for the ^1^H NMR shielding in Figure 1a shows that the main shielding contribution in the molecular plane originates from the outer regions of the molecular electron density, where the diatropic ring current is strong.^11,33^ Deshielding contributions arise close to the hydrogen nucleus and close to its adjacent (*ipso*) carbon. Shielding and deshielding contributions also arise from the valence electrons of the *ipso* and the nearest-neighbor (*ortho*) carbon atoms due to their local atomic current-density fluxes. All carbons have both shielding and deshielding contributions for ^1^H arising from the core electrons due to atomic current densities around the nucleus.

The two core contributions cancel almost completely because the atomic current density has the same strength on both sides of the nucleus, and the relative distance to the positive (shielding, blue) and negative (deshielding, red) areas from the studied hydrogen nucleus is almost the same for the carbon atoms in the *meta* and *para* positions.

The *zz* contribution to the magnetic shielding density in the molecular plane for a ^13^C nucleus in Figure 1b has an onion-like shell structure of shielding and deshielding contributions. The shielding contribution close to the nucleus arises from the core electrons, whereas the valence electrons deshield the nucleus. In the next shell, the shielding contribution originates from the diatropic ring current that flows on the outer side of the molecular ring near the hydrogen as well as from the paratropic ring current inside the C_6_H_6_ ring. The atomic current density in the valence orbitals of the *ortho* carbon atoms also contributes to the ^13^C shielding on closer side of the *ortho* carbon, while the contributions are deshielding on the remote side.

**Figure 2:**
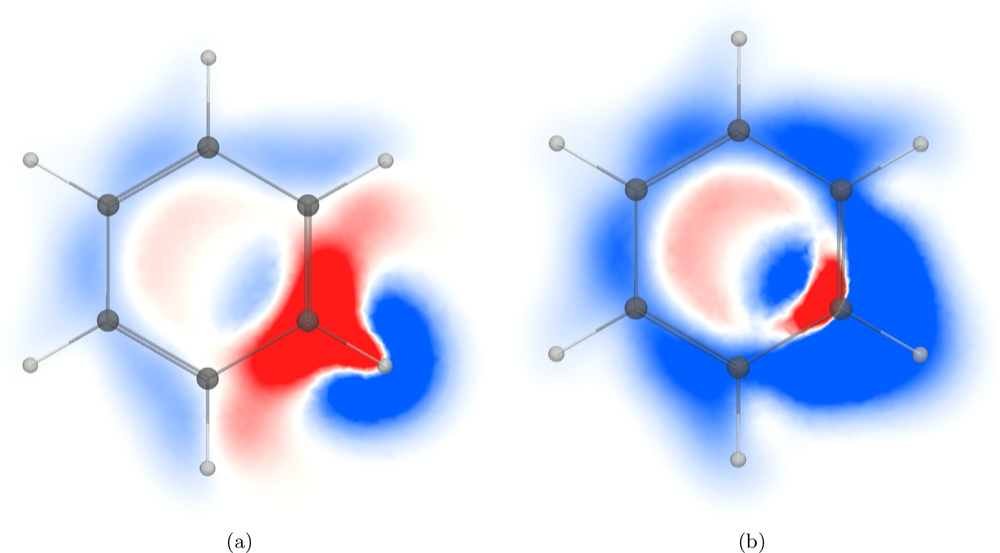
*zz* component of the magnetic shielding density of the (a) ^1^H NMR shielding and (b) ^13^C NMR shielding of C_6_H_6_ calculated 1 *a*_0_ above the molecular plane. The shielding contributions are shown in blue and the deshielding contributions in red in the range [−0.2; 0.2].

The ring-current contribution to the nuclear magnetic shielding constants can be analyzed by plotting the spatial distribution of the σ_*zz*_ component to the nuclear magnetic shielding density. The *zz* component of the ^1^H NMR shielding density calculated in a plane 1 *a*_0_ above the molecular plane is shown in Figure 2a. The diatropic ring current flowing on the outside of the hydrogen shields the hydrogen nucleus. The ring current on the other side of the ring also shields it, while the diatropic ring current flowing on the inside of the hydrogen is deshielding. The paratropic ring current inside the C_6_H_6_ ring deshields the hydrogen nucleus on the remote half of the ring, whereas inside the *ipso* carbon atom the paratropic ring current shields the hydrogen nucleus. The sign of the shielding contributions depends on the direction of the current density with respect to the studied nucleus according to the Biot–Savart expression in eq 12.

The ring-current contribution to the ^13^C NMR shielding is seen in Figure 2b, where shielding contributions appear along the outer perimeter of the carbon ring. The paratropic ring current inside the C_6_H_6_ ring leads to a shielding contribution near the studied carbon atom, whereas it is deshielding on the remote interior part of the ring. The deshielding contribution in the vicinity of the studied carbon originates from the diatropic ring current passing on the inside of the carbon atom.

**Figure 3:**
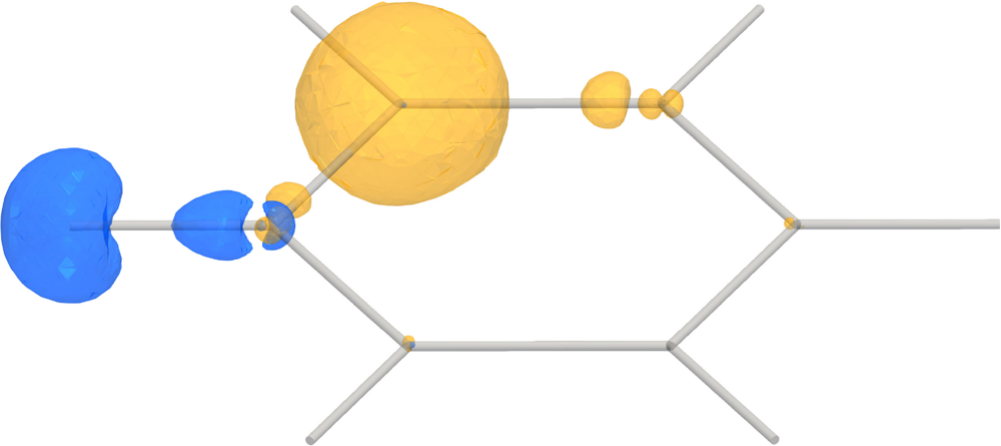
Absolute value of the magnetic shielding density of the ^1^H NMR shielding (blue) and ^13^C NMR shielding (yellow) in C_6_H_6_ represented as contours with isovalue 4.8. The *ipso* atoms have the largest contributions.

The absolute value of the nuclear magnetic shielding density is illustrated by using a contour surface in Figure 3, where blue represents the shielding density of the hydrogen atom, while yellow is used to illustrate the shielding of the carbon atom. Figure 3 reveals that the shielding density near the *ortho* atoms contributes significantly, whereas the more distant atoms have negligible contributions, as expected due to the |**r** – **R**_*I*_|^–3^ denominator in the vector potential of **m**_*I*_.

Atomic contributions to the isotropic nuclear magnetic shielding constants can be analyzed by integration over atomic subdomains, yielding a compact representation of the spatial distribution of the shielding density. In this work, the atomic subdomains are defined by the Becke partitioning,^36^ as discussed in section 2.2. Even though Becke partitioning was originally aimed for efficient numerical integration of density functionals, it has been shown to be useful for e.g. constructing mathematically well-based Pipek–Mezey orbital localization techniques,^51,52^ and with a careful choice of the partitioning function it yields chemically sound atomic charges and bond orders.^53^ The decomposition depends on the partitioning, i.e., the choice for the atomic weight functions. The original Becke partitioning yields a rough idea of the atomic decomposition of the shielding density; more sophisticated atomic decompositions are left to further work.

The resulting atomic contributions to the ^1^H NMR and ^13^C NMR magnetic shieldings of C_6_H_6_ are given in Tables 1 and 2, respectively. These data suggest that the main contributions to the shielding originate from the vicinity of the studied atom and its nearest neighbors, which is utilized when using local methods to calculate nuclear magnetic shielding constants.^21,22^

**Table 1:** Atomic Contributions to the ^1^H NMR Shielding of C_6_H_6_ Calculated at the B3LYP/def2-TZVP Level of Theory

domain	total	positive	negative	percentage
*ipso* C^a^	1.48	5.24	–3.76	6.11%
*ortho* C	0.64	1.43	–0.79	2.64%
*meta* C	0.52	0.77	–0.25	2.16%
*para* C	0.42	0.63	–0.21	1.74%
*ipso* H^b^	18.97	20.33	–1.36	78.13%
*ortho* H	0.29	0.36	–0.07	1.19%
*meta* H	0.17	0.17	–0.00	0.71%
*para* H	0.15	0.15	–0.00	0.61%
total	24.28	31.83	–7.54	100.00%

a *Ipso* C is the carbon connected to the studied hydrogen nucleus.

b *Ipso* H is the studied hydrogen nucleus.

**Table 2:** Atomic Contributions to the ^13^C NMR Shielding of C_6_H_6_ Calculated at the B3LYP/def2-TZVP Level of Theory

domain	total	positive	negative	percentage
*ipso* C^a^	35.17	107.15	–71.97	70.49%
*ortho* C	3.49	4.96	–1.47	7.00%
*meta* C	1.04	1.56	–0.52	2.08%
*para* C	0.74	1.19	–0.45	1.48%
*ipso* H^b^	2.80	2.81	–0.01	5.61%
*ortho* H	0.65	0.66	–0.01	1.30%
*meta* H	0.30	0.30	–0.00	0.59%
*para* H	0.24	0.24	–0.00	0.49%
total	49.90	126.35	–76.44	100.00%

a *Ipso* C is the studied carbon nucleus.

b *Ipso* H is the hydrogen connected to the studied carbon nucleus.

The contribution to the ^1^H NMR shielding from the atomic domain of the studied hydrogen is 78.13% of the total shielding, while the contribution assigned to each *ipso* carbon is 6.11%. Contributions from all other atoms are in the interval of [0.61, 2.64]%. The contribution to the ^13^C NMR shielding from the studied carbon is 70.49%. The *ipso* hydrogen and *ortho* carbons contribute with 5.61% and 7.00%, respectively, whereas the ^13^C NMR contributions from the rest of the atoms are in the interval of [0.49, 2.08]%.

As a side note, although the molecular structure of borazine (B_3_N_3_H_6_) is similar to that of benzene, a previous study of shielding densities suggested that borazine is nonaromatic.^15^ However, a follow-up study showed that B_3_N_3_H_6_ does sustain a diatropic ring current, although its strength is only 25% of that in C_6_H_6_.^54^ A comparison of the *zz* contribution to the shielding densities of C_6_H_6_ and B_3_N_3_H_6_ (shown in the Supporting Information) reveals that B_3_N_3_H_6_ has a similar but weaker ring-current contribution to the shielding density as for C_6_H_6_. Thus, B_3_N_3_H_6_ cannot be considered to be nonaromatic.

### 3.2 Cyclobutadiene

**Figure 4:**
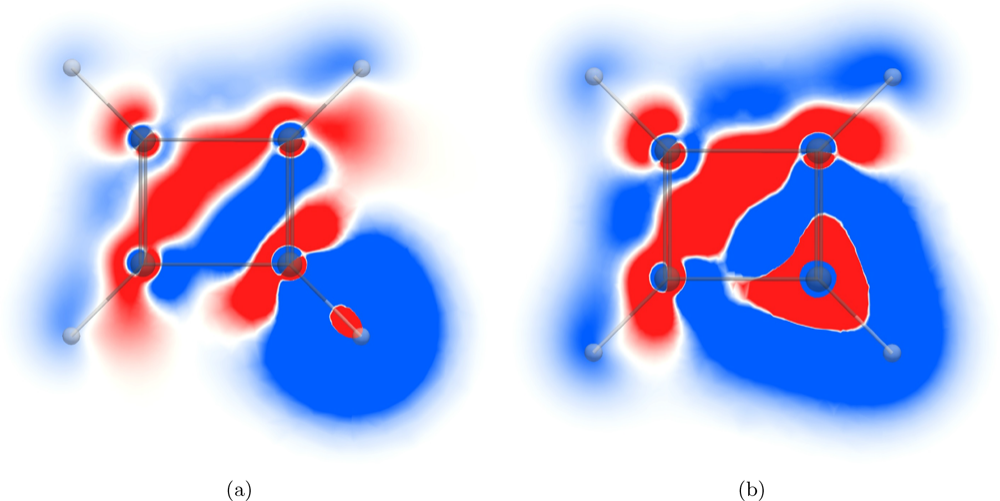
*zz* component of the magnetic shielding density of the (a) ^1^H NMR shielding and (b) ^13^C NMR shielding in the molecular plane of C_4_H_4_. The shielding contribution is shown in blue and the deshielding contribution in red in the range [−0.2; 0.2].

The *zz* contribution to the ^1^H NMR shielding density in the molecular plane of C_4_H_4_ shown in Figure 4a is similar to the one for C_6_H_6_ in Figure 1a. Even though C_4_H_4_ is antiaromatic, it sustains a diatropic ring current along the outer edge of the molecule outside the hydrogen giving rise to a similar shielding contribution outside the hydrogen like in C_6_H_6_.^33^ The ring current is paratropic inside the ring as in C_6_H_6_. Deshielding contributions appear at the hydrogen nucleus as well as at the *ipso* and *ortho* carbons due to local current densities. The atomic current density in the core of the carbon atoms leads to shielding and deshielding contributions that practically cancel, as for C_6_H_6_.

The contributions to the ^13^C magnetic shielding density in the molecular plane of C_4_H_4_ in Figure 4b also remind of those for C_6_H_6_. The onion structure of the alternating shielding and deshielding contributions around the studied carbon atom originates from current densities with different flux directions in the vicinity of the atom. The diatropic atomic current density in its core orbitals, the diatropic ring current flowing on the outside of hydrogen atom, and the paratropic ring current inside the C_4_H_4_ ring shield the carbon nucleus, whereas the atomic current density of the valence orbitals deshields it.

**Figure 5:**
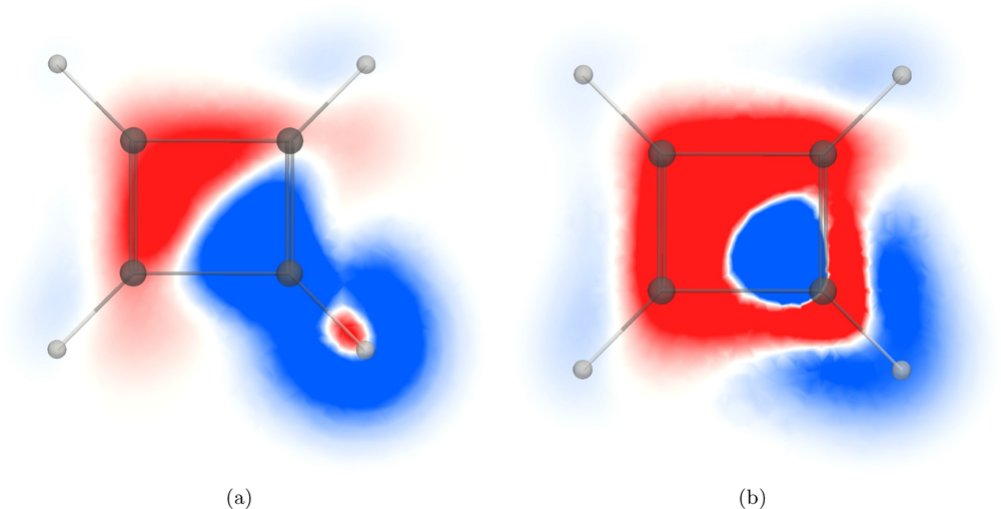
*zz* component of the magnetic shielding density of the (a) ^1^H NMR shielding and (b) ^13^C NMR shielding of C_4_H_4_ calculated 1 *a*_0_ above the molecular plane. The shielding contribution is shown in blue and the deshielding contribution in red in the range [−0.2; 0.2] in (b).

In contrast, the magnetic shielding density in a plane 1 *a*_0_ above (or below) the molecular plane of C_4_H_4_ differs completely from the one for C_6_H_6_ because C_6_H_6_ sustains a diatropic ring current in the π orbitals, while the current density of C_4_H_4_ is paratropic there. The ^1^H and ^13^C magnetic shielding densities of C_4_H_4_ in Figures 5a and 5b show that the diatropic ring current along the outer edge of the molecule leads to a shielding contribution to ^1^H NMR and ^13^C NMR shieldings. The strong paratropic ring current which resides mainly inside the molecular ring leads to a shielding contribution to ^1^H NMR in the closer half of the ring and a deshielding contribution from the remote part of the ring due to the different directions of the current-density fluxes relative to the studied hydrogen nucleus.

The deshielding contribution to the ^13^C NMR shielding from the paratropic ring current dominates above the ring on the inside of it. A small shielding area is seen in Figure 5b, where the relative direction of the paratropic ring current leads to magnetic shielding. The paratropic ring current on the outside of the studied carbon deshields the carbon nucleus. The diatropic ring current along the outer edge of the molecule results in a weak shielding contribution in the vicinity of the hydrogen atom.

**Figure 6:**
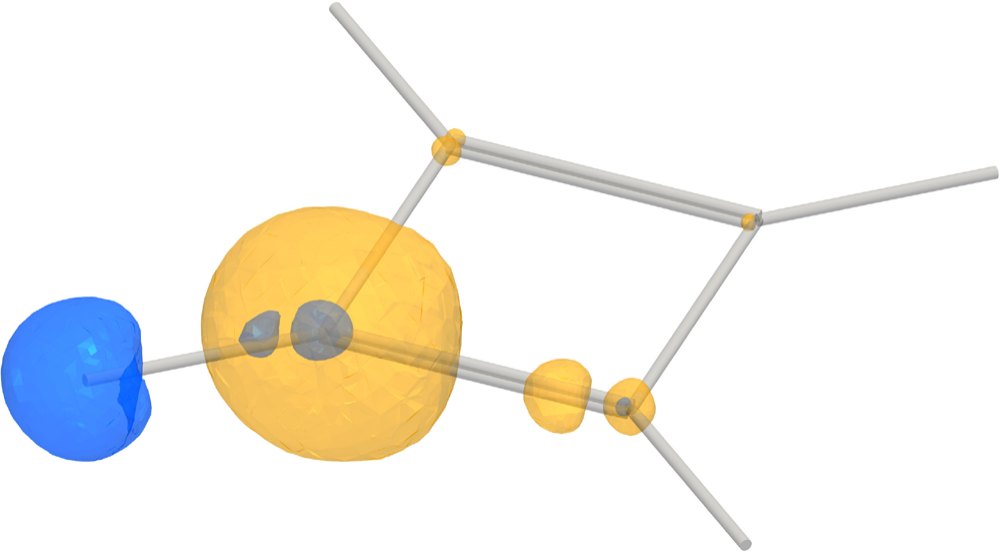
Absolute value of the magnetic shielding density of the ^1^H NMR shielding (blue) and ^13^C NMR shielding (yellow) in C_4_H_4_ represented as contours with isovalue 7. The *ipso* atoms have the largest contributions.

The absolute value of the nuclear magnetic shielding density is illustrated by using a contour surface in Figure 6, again showing that the most significant contributions arise from the *ipso* atoms, with some contributions from the *ortho* atoms.

The atomic contributions to the ^1^H NMR and ^13^C NMR magnetic shieldings of C_4_H_4_ are given in Tables 3 and 4, respectively. Table 3 shows that 70.98% of the ^1^H NMR shielding of C_4_H_4_ originates from the atomic domain of the studied hydrogen. The contribution from the *ipso* carbon is 24.98%. The rest of the atoms contribute with less than 3.66%. The contributions from the *para* carbon and the *ortho* carbon with a formal single bond to the studied carbon are even negative.

**Table 3:** Atomic Contributions to the ^1^H NMR Shielding of C_4_H_4_ Calculated at the B3LYP/def2-TZVP Level of Theory

domain	total	positive	negative	percentage
*ipso* C^a^	6.48	8.52	–2.05	24.98%
*ortho* C^b^	–0.15	0.81	–0.97	–0.59%
*ortho* C^c^	0.95	1.83	–0.88	3.66%
*para* C	–0.44	0.42	–0.87	–1.71%
*ipso* H^a^	18.41	19.47	–1.06	70.98%
*ortho* H^b^	0.22	0.24	–0.03	0.83%
*ortho* H^c^	0.30	0.32	–0.02	1.17%
*para* H	0.18	0.18	–0.00	0.70%
total	25.93	31.80	–5.86	100.00%

a *Ipso* is the studied atom or its nearest neighbor.

b Moiety with a single bond to the *ipso* carbon.

c Moiety with a double bond to the *ipso* carbon.

**Table 4:** Atomic Contributions to the ^13^C NMR Shielding of C_4_H_4_ Calculated at the B3LYP/def2-TZVP Level of Theory

domain	total	positive	negative	percentage
*ipso* C^a^	32.51	106.93	–74.41	87.50%
*ortho* C^b^	–0.73	1.74	–2.47	–1.96%
*ortho* C^c^	2.66	5.38	–2.71	7.17%
*para* C	–1.16	0.92	–2.09	–3.13%
*ipso* H^a^	2.49	2.51	–0.03	6.69%
*ortho* H^b^	0.45	0.46	–0.01	1.22%
*ortho* H^c^	0.61	0.61	–0.00	1.64%
*para* H	0.32	0.33	–0.00	0.87%
total	37.16	118.90	–81.74	100.00%

a *Ipso* is the studied atom or its nearest neighbor.

b Moiety with a single bond to the *ipso* carbon.

c Moiety with a double bond to the *ipso* carbon.

The contribution to the ^13^C NMR shielding from the studied carbon is 87.50%. The *ipso* hydrogen contributes with 6.69%, and the *ipso* carbon with a formal double bond to the studied carbon contributes with 7.71%. Contributions to ^13^C NMR from the rest of the atoms are small. The contributions from the *ortho* carbon with a formal single bond to the studied carbon and the carbon in the *para* position are also in this case negative.

### 3.3 Basis Set Dependence

We investigated the basis set truncation error in the def2-TZVP basis set with additional calculations using the fully uncontracted pc-*n* (unpc-*n*) polarization consistent basis sets series^55^ and their augmented versions.^56^ The basis set study was performed with Gaussian,^57^ and all basis sets were obtained from the Basis Set Exchange.^58^ The full set of results is shown in the Supporting Information.

The resulting B3LYP complete basis set estimates from the quintuple-ζ unpc-4 set, which has a 11s6p3d2f1g and 18s11p6d3f2g1h composition for H and C, respectively, were found to be 42.35 and 24.03 ppm for the ^13^C and ^1^H NMR shieldings, respectively, for C_6_H_6_. For C_4_H_4_, the shieldings are 30.15 and 25.72 ppm, respectively. The def2-TZVP values for ^13^C in C_6_H_6_ and C_4_H_4_ are 49.90 and 37.16 ppm, which are 13.13 and 7.01 ppm from the unpc-4 values. The ^1^H NMR shieldings of 24.28 and 25.93 ppm agree well with the unpc-4 values, with differences of just 0.25 and 0.21 ppm.

Because of the noticeable basis set truncation error for the carbon shieldings, additional calculations were performed with the generally contracted pc-*n* basis sets,^55^ their newer versions based on segmented contractions^59^ (pcseg-*n*) and specializations thereof to the reproduction of nuclear magnetic shieldings^60^ (pcSseg-*n*), as well as with the Karlsruhe def2 family of basis sets.^43^ The pcseg-3 basis set^59^ was found to yield excellent agreement with the unpc-4 values: the pcseg-3 basis set yields ^13^C and ^1^H shieldings of 30.09 and 25.64 ppm for C_4_H_4_ and 42.89 and 23.96 ppm for C_6_H_6_, respectively.

Because of the good accuracy of the pcseg-3 basis set, spatial decompositions for C_4_H_4_ and C_6_H_6_ were recomputed in this basis; the decompositions are shown in the Supporting Information. Comparison of these data to the values in Tables 2 and 4 shows that basis set truncation error in def2-TZVP significantly affects only the shielding contribution from the *ipso* carbon, while the shielding contributions from the other atoms are strikingly similar, differing only up to 0.05 ppm for C_4_H_4_ and 0.03 ppm for C_6_H_6_. This strongly suggests that the differences originate from orbitals localized to the *ipso* carbon, that is, an insufficient flexibility in the semicore region of the def2-TZVP basis set of carbon. Because the truncation error changes significantly the absolute nuclear magnetic shielding of the studied carbon, this also affects the relative percentages of the atomic contributions. Similar conclusions can also be made for the hydrogen shieldings by comparison of the data in the Supporting Information to Tables 1 and 3: the largest change (0.27 ppm) originates from the *ipso* hydrogen, while the contributions from all other atoms are negligible: less than 0.05 ppm for C_6_H_6_ and less than 0.03 ppm for C_4_H_4_.

## 4 Summary and Conclusions

We have implemented methods for calculating and visualizing nuclear magnetic shielding densities in the GIMIC program. Studies of the shielding densities of benzene (C_6_H_6_) and cyclobutadiene (C_4_H_4_) show that the direction of the current-density flux relative to the studied nucleus determines whether the current density shields or deshields the nuclear magnetic moment. The paratropic ring current in the molecular plane within the C_6_H_6_ and C_4_H_4_ rings shields the studied nucleus when the current flows in the vicinity of the nucleus, while the current becomes deshielding on the remote side of the ring. The paratropic ring current inside the ring is much weaker in the aromatic benzene molecule than in the antiaromatic cyclobutadiene molecule. Benzene sustains a strong diatropic ring current in the π orbitals above and below the molecular ring, which results in shielding contributions to the ^1^H NMR and ^13^C NMR shieldings. However, the ring current passing the *ipso* carbon deshields the ^1^H nuclear magnetic moment because it is a diatropic ring current near the studied ^1^H nucleus that flows on the inside of it. The same holds for the ^13^C NMR shielding. However, the diatropic ring current passing on the inside of the carbon is weaker and leads only to a small deshielding contribution.

The ^1^H NMR and ^13^C NMR shielding densities in the molecular plane of C_4_H_4_ are similar to the ones of C_6_H_6_, whereas 1 *a*_0_ from the molecular plane the shielding densities are completely different. C_4_H_4_ sustains a strong paratropic ring current in the π orbitals inside the ring, whereas the ring current in C_6_H_6_ is diatropic and flows mainly on the outside of the carbon ring.

Calculations of atomic contributions to the nuclear magnetic shielding constants using Becke’s partitioning show that the largest contributions originate from the *ipso* atoms and its nearest neighbors. The *ipso* carbon contributes with 70.49% and 87.50% to the ^13^C NMR shielding of C_6_H_6_ and C_4_H_4_, respectively. The contribution from the *ipso* hydrogen to the ^1^H NMR shielding is 78.13% and 70.98% for C_6_H_6_ and C_4_H_4_, respectively. Even for small molecules like C_4_H_4_ and C_6_H_6_, contributions from more distant atoms are only a few percent, which is utilized in local methods to calculate nuclear magnetic shielding constants.

Although the B3LYP/def2-TZVP level of theory was used for the most part of the present work, the methods presented herein can also be used with larger basis sets and post-Hartree–Fock levels of theory. We repeated the analysis in the pcseg-3 basis set, which we found to yield shielding constants in good agreement with our complete basis set estimates, which showed that most of the deficiencies in the def2-TZVP data originate from the atom under study, while the contributions from all other atomic domains are essentially already converged in def2-TZVP.

## Supporting Information Available

B3LYP/def2-TZVP optimized molecular structures for C_4_H_4_, C_6_H_6_, and B_3_N_3_H_6_, atomic shielding contributions calculated by using the pcseg-3 basis set, a basis set study, and the shielding density of borazine (PDF)
