# Supplementary material for: Spatial Contributions to Nuclear Magnetic Shieldings
Source: J Phys Chem A. 2021 Feb 19;125(8):1778–86. doi: 10.1021/acs.jpca.0c10884 (PMC8023705; doi:10.1021/acs.jpca.0c10884)
Supplement: Supplementary file 1 — jp0c10884_si_001.pdf [file jp0c10884_si_001.pdf]

Supporting Information:
Spatial Contributions to Nuclear Magnetic Shieldings

Rahul Kumar Jinger,[†] Heike Fliegl,[‡] Radovan Bast,[¶] Maria Dimitrova,[§] Susi Lehtola,[§,‖] and Dage Sundholm*,[§]

†*Indian Institute of Science Education and Research, Dr. Homi Bhabha Road, Pashan, Pune 411008, India*

‡*Karlsruhe Institute of Technology, Institute of Nanotechnology, Hermann-von-Helmholtz Platz 1, D-76344 Eggenstein-Leopoldshafen, Germany*

¶*UiT Arctic University Norway, Department Information Technology, Tromsø, Norway*

§*Department of Chemistry, P.O. Box 55 (A.I. Virtanens plats 1), FI-00014 University of Helsinki, Finland*

‖*Molecular Sciences Software Institute, Blacksburg, Virginia 24061, United States*

E-mail: dage.sundholm@helsinki.fi

Optimized molecular geometries

Benzene

```
12
B3LYP/def2-TZVP optimized geometry in angstrom
C     1.2031019   -0.6946112    0.0000000
C     1.2031019    0.6946112    0.0000000
C    -0.0000000    1.3892224    0.0000000
C    -1.2031019    0.6946112    0.0000000
C    -1.2031019   -0.6946112    0.0000000
C    -0.0000000   -1.3892224    0.0000000
H     2.1366969   -1.2336225    0.0000000
H     2.1366969    1.2336225    0.0000000
H    -0.0000000    2.4672450    0.0000000
H    -2.1366969    1.2336225    0.0000000
H    -2.1366969   -1.2336225    0.0000000
H    -0.0000000   -2.4672450    0.0000000
```

The Turbomole optimized benzene geometry as an xyz file[S1]

Cyclobutadiene

```
8
B3LYP/def2-TZVP optimized geometry in angstrom
C     0.7832091   -0.6628760    0.0000000
H     1.5452463   -1.4272184    0.0000000
C     0.7832091    0.6628760    0.0000000
C    -0.7832091    0.6628760    0.0000000
C    -0.7832091   -0.6628760    0.0000000
H     1.5452463    1.4272184    0.0000000
H    -1.5452463    1.4272184    0.0000000
H    -1.5452463   -1.4272184    0.0000000
```

The Turbomole optimized cyclobutadiene geometry as an xyz file.

Borazine

```
12
B3LYP/def2-TZVP optimized geometry in angstrom
H    -1.3207798   -2.2876577    0.0000000
B    -0.7243343   -1.2545839    0.0000000
N     0.7031349   -1.2178653    0.0000000
N    -1.4062697    0.0000000    0.0000000
B    -0.7243343    1.2545839    0.0000000
H    -2.4132950    0.0000000    0.0000000
H    -1.3207798    2.2876577    0.0000000
N     0.7031349    1.2178653    0.0000000
H     1.2066475   -2.0899748    0.0000000
B     1.4486687    0.0000000    0.0000000
H     2.6415596    0.0000000    0.0000000
H     1.2066475    2.0899748    0.0000000
```

The Turbomole optimized borazine geometry as an xyz file.

Basis set convergence of shielding constants

The basis set convergence fo the B3LYP shielding constants was studied using GAUSSIAN[S2] with a (99,590) quadrature grid with various basis sets discussed in the main text at the fixed B3LYP/def2-TZVP molecular geometries. The results are shown in tables S1 and S2 for C_6H_6 and C_4H_4, respectively. Comparing the aug-pc-n and pc-n data shows

that diffuse functions do not affect the shieldings, while the small differences between the quadruple-ζ un-pc-3 data and the quintuple-ζ un-pc-4 data (0.2 ppm for carbon, 0.02 ppm for hydrogen) suggests that the un-pc-4 values are close to the complete basis set limit.

Atomic contributions to magnetic shielding constants in pcseg-3 basis

Benzene

The atomic contributions to the ^{1}H and ^{13}C magnetic shielding constants of C_6H_6 are shown in tables S3 and S4, respectively.

Cyclobutadiene

The atomic contributions to the ^{1}H and ^{13}C magnetic shielding constants of C_4H_4 are shown in tables S5 and S6, respectively.

Table S1: ^{13}C and ^{1}H nuclear magnetic shielding constants in ppm for C_6H_6 for the B3LYP functional and various basis sets. The first part of the table shows data for uncontracted basis sets, and the second part data for contracted basis sets. Also the number of basis functions N_{bf} is shown.

Basis set	Carbon	Hydrogen	N_{bf}
un-pc-0	79.942	25.637	102
un-pc-1	57.800	24.450	186
un-pc-2	44.839	24.174	372
un-pc-3	42.565	24.048	732
un-pc-4	42.354	24.032	1188
un-aug-pc-0	78.475	25.680	132
un-aug-pc-1	56.955	24.449	264
un-aug-pc-2	44.671	24.143	522
un-aug-pc-3	42.556	24.045	978
un-aug-pc-4	42.353	24.031	1554
pc-0	76.906	25.643	66
pc-1	59.680	24.460	114
pc-2	46.291	24.150	264
pc-3	43.129	24.049	588
pc-4	42.204	24.032	1032
pcseg-0	81.281	26.027	66
pcseg-1	59.653	24.458	114
pcseg-2	46.345	24.160	264
pcseg-3	43.608	24.042	558
pcseg-4	43.193	24.030	972
pcSseg-0	52.665	25.965	66
pcSseg-1	46.701	24.291	132
pcSseg-2	42.982	24.103	300
pcSseg-3	42.307	24.030	630
pcSseg-4	42.265	24.029	1044
def2-SVP	66.724	24.559	114
def2-TZVP	50.601	24.367	222
def2-TZVPP	50.560	24.114	270
def2-QZVP	46.152	24.096	522

Table S2: ^{13}C and ^{1}H nuclear magnetic shielding constants in ppm for C_4H_4 for the B3LYP functional and various basis sets. The first part of the table shows data for uncontracted basis sets, and the second part data for contracted basis sets. Also the number of basis functions N_{bf} is shown.

Basis set	Carbon	Hydrogen	N_{bf}
un-pc-0	65.463	26.953	68
un-pc-1	44.882	26.110	124
un-pc-2	32.614	25.838	248
un-pc-3	30.352	25.737	488
un-pc-4	30.149	25.722	792
un-aug-pc-0	64.428	26.722	88
un-aug-pc-1	44.583	26.044	176
un-aug-pc-2	32.477	25.810	348
un-aug-pc-3	30.343	25.734	652
un-aug-pc-4	30.149	25.721	1036
pc-0	62.524	26.986	44
pc-1	47.834	26.124	76
pc-2	33.955	25.825	176
pc-3	30.808	25.736	392
pc-4	30.001	25.722	688
pcseg-0	67.057	27.222	44
pcseg-1	47.824	26.119	76
pcseg-2	34.038	25.828	176
pcseg-3	31.332	25.730	372
pcseg-4	30.925	25.719	648
pcSseg-0	37.974	27.184	44
pcSseg-1	33.577	25.955	88
pcSseg-2	30.772	25.771	200
pcSseg-3	30.095	25.718	420
pcSseg-4	30.061	25.719	696
def2-SVP	55.446	26.229	76
def2-TZVP	38.338	26.022	148
def2-TZVPP	38.240	25.826	180
def2-QZVP	33.862	25.787	348

Table S3: Atomic contributions to the ^{1}H NMR shielding of C_6H_6 calculated at the B3LYP/pcseg-3 level of theory.

Domain	Total	Positive	Negative	Percentage
ipso C[a]	1.43	5.17	-3.74	5.99 %
ortho C	0.63	1.42	-0.78	2.64 %
meta C	0.52	0.77	-0.25	2.16 %
para C	0.42	0.63	-0.21	1.75 %
ipso H[b]	18.70	20.48	-1.77	78.08 %
ortho H	0.30	0.37	-0.07	1.24 %
meta H	0.17	0.18	-0.00	0.73 %
para H	0.15	0.15	-0.00	0.63 %
Total	23.96	31.89	-7.93	100.00 %

[a] *ipso* C is the carbon connected to the studied hydrogen nucleus.

[b] *ipso* H is the studied hydrogen nucleus.

Table S4: Atomic contributions to the ^{13}C NMR shielding of C_6H_6 calculated at the B3LYP/pcseg-3 level of theory.

Domain	Total	Positive	Negative	Percentage
ipso C[a]	28.18	102.01	-73.83	65.70 %
ortho C	3.46	4.94	-1.48	8.08 %
meta C	1.03	1.55	-0.53	2.39 %
para C	0.73	1.18	-0.45	1.69 %
ipso H[b]	2.83	2.84	-0.01	6.61 %
ortho H	0.66	0.67	-0.01	1.53 %
meta H	0.30	0.30	-0.00	0.70 %
para H	0.25	0.25	-0.00	0.58 %
Total	42.89	121.21	-78.32	100.00 %

[a] *ipso* C is the studied carbon nucleus.
[b] *ipso* H is the hydrogen connected to the studied carbon nucleus.

Table S5: Atomic contributions to the ^{1}H magnetic shielding of C_4H_4 calculated at the B3LYP/pcseg-3 level of theory.

Domain	Total	Positive	Negative	Percentage
ipso C[a]	6.48	8.52	-2.04	25.28 %
ortho C[b]	-0.18	0.80	-0.98	-0.70 %
ortho C[c]	0.95	1.83	-0.88	3.70 %
para C	-0.45	0.42	-0.88	-1.78 %
ipso H[a]	18.14	19.53	-1.39	70.73 %
ortho H[b]	0.22	0.24	-0.03	0.84 %
ortho H[c]	0.31	0.32	-0.01	1.21 %
para H	0.18	0.18	-0.00	0.71 %
Total	25.64	31.85	-6.21	100.00 %

[a] *ipso* is the studied atom or its nearest neighbor.
[b] moiety with a single bond to the *ipso* carbon.
[c] moiety with a double bond to the *ipso* carbon.

Table S6: Atomic contributions to the ^{13}C magnetic shielding of C_4H_4 calculated at the B3LYP/pcseg-3 level of theory.

Domain	Total	Positive	Negative	Percentage
ipso C[a]	25.53	102.00	-76.47	84.84 %
ortho C[b]	-0.78	1.72	-2.50	-2.59 %
ortho C[c]	2.63	5.36	-2.73	8.77 %
para C	-1.19	0.91	-2.10	-3.96 %
ipso H[a]	2.49	2.52	-0.03	8.29 %
ortho H[b]	0.45	0.47	-0.01	1.51 %
ortho H[c]	0.62	0.62	-0.00	2.05 %
para H	0.33	0.33	-0.00	1.09 %
Total	30.09	113.93	-83.84	100.00 %

[a] *ipso* is the studied atom or its nearest neighbor.
[b] moiety with a single bond to the *ipso* carbon.
[c] moiety with a double bond to the *ipso* carbon.

Magnetic shielding densities for borazine

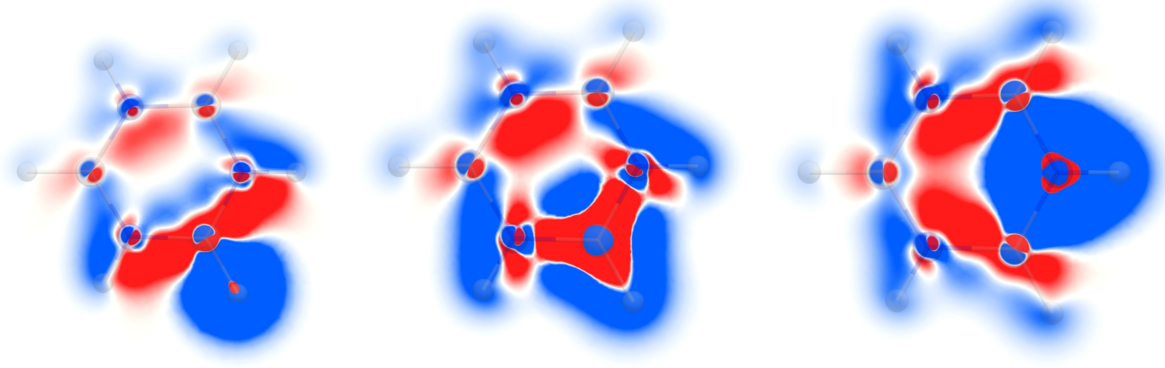

(a) (b) (c)

Figure S1: The trace of the magnetic shielding density of the S1(a) ^{1}H NMR shielding, S1(b) ^{11}B NMR shielding, and S1(c) ^{15}N NMR shielding of $B_3N_3H_6$ calculated in the molecular plane. The shielding contributions are shown in blue and the deshielding contributions in red in the range of $[-0.2; 0.2]$.

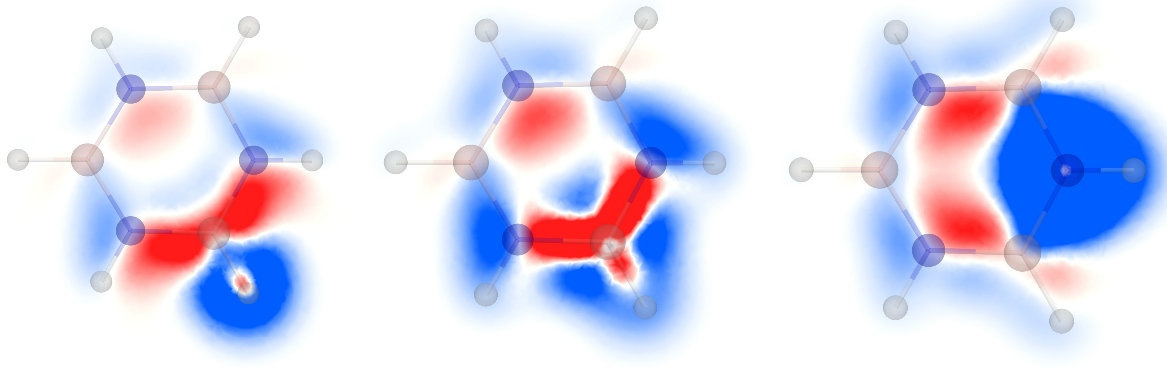

(a) (b) (c)

Figure S2: The zz component of the magnetic shielding density of the S2(a) ^{1}H NMR shielding, S2(b) ^{11}B NMR shielding, and S2(c) ^{15}N NMR shielding of $B_3N_3H_6$ calculated $1\,a_0$ above the molecular plane. The shielding contributions are shown in blue and the deshielding contributions in red in the range of $[-0.2; 0.2]$.

References

(S1) Balasubramani, S. G.; Chen, G. P.; Coriani, S.; Diedenhofen, M.; Frank, M. S.; Franzke, Y. J.; Furche, F.; Grotjahn, R.; Harding, M. E.; Hättig, C.; Hellweg, A.; Helmich-Paris, B.; Holzer, C.; Huniar, U.; Kaupp, M.; Marefat Khah, A.; Karbalaei Khani, S.; Müller, T.; Mack, F.; Nguyen, B. D.; Parker, S. M.; Perlt, E.; Rappoport, D.; Reiter, K.; Roy, S.; Rückert, M.; Schmitz, G.; Sierka, M.; Tapavicza, E.; Tew, D. P.; van Wüllen, C.; Voora, V. K.; Weigend, F.; Wodyński, A.; Yu, J. M. TURBOMOLE: Modular program suite for ab initio quantum-chemical and condensed-matter simulations. *J. Chem. Phys.* **2020**, *152*, 184107.

(S2) Frisch, M. J.; Trucks, G. W.; Schlegel, H. B.; Scuseria, G. E.; Robb, M. A.; Cheeseman, J. R.; Zakrzewski, V. G.; Montgomery Jr, J. A.; Stratmann, R. E.; Burant, J. C.; Dapprich, S.; Millam, J. M.; Daniels, A. D.; Kudin, K. N.; Strain, M. C.; Farkas, O.; Tomasi, J.; Barone, V.; Cossi, M.; Cammi, R.; Mennucci, B.; Pomelli, C.; Adamo, C.; Clifford, S.; Ochterski, J.; Petersson, G. A.; Ayala, P. Y.; Cui, Q.; Morokuma, K.; Malick, D. K.; Rabuck, A. D.; Raghavachari, K.; Foresman, J. B.; Cioslowski, J.; Ortiz, J. V.; Baboul, A. G.; Stefanov, B. B.; Liu, G.; Liashenko, A.; Piskorz, P.; Komaromi, I.; Gomperts, R.; Martin, R. L.; Fox, D. J.; Keith, T.; Al-Laham, M. A.; Peng, C. Y.; Nanayakkara, A.; Gonzalez, C.; Challacombe, M.; Gill, P. M. W.; Johnson, B.; Chen, W.; Wong, M. W.; Andres, J. L.; Gonzalez, C.; Head-Gordon, M.; Replogle, E. S.; Pople, J. A. Gaussian 09, Revision A.02, Gaussian, Inc., Pittsburgh PA. 2016.
